# Cardioprotective effects of lixisenatide in rat myocardial ischemia-reperfusion injury studies

**DOI:** 10.1186/1479-5876-11-84

**Published:** 2013-03-28

**Authors:** Paulus Wohlfart, Wolfgang Linz, Thomas Hübschle, Dominik Linz, Jochen Huber, Sibylle Hess, Daniel Crowther, Ulrich Werner, Hartmut Ruetten

**Affiliations:** 1 Sanofi R&D Diabetes Division, Industriepark Hoechst, H825, 65926, Frankfurt/Main, Germany; 2 Faculty of Medicine, Saarland University, Saarbrücken, Homburg/Saar, Germany; 3 Current Adress: Boehringer-Ingelheim Pharma GmbH, CMDR, Biberach a.d.R., Germany

**Keywords:** Lixisenatide, GLP1-receptor agonist, Diabetes, Macrovascular risk, Cardioprotection, Myocardial ischemia-reperfusion, Cardiac dysfunction, Pre-clinical models

## Abstract

**Background:**

Lixisenatide is a glucagon-like peptide-1 analog which stimulates insulin secretion and inhibits glucagon secretion and gastric emptying. We investigated cardioprotective effects of lixisenatide in rodent models reflecting the clinical situation.

**Methods:**

The acute cardiac effects of lixisenatide were investigated in isolated rat hearts subjected to brief ischemia and reperfusion. Effects of chronic treatment with lixisenatide on cardiac function were assessed in a modified rat heart failure model after only transient coronary occlusion followed by long-term reperfusion. Freshly isolated cardiomyocytes were used to investigate cell-type specific mechanisms of lixisenatide action.

**Results:**

In the acute setting of ischemia-reperfusion, lixisenatide reduced the infarct-size/area at risk by 36% ratio without changes on coronary flow, left-ventricular pressure and heart rate. Treatment with lixisenatide for 10 weeks, starting after cardiac ischemia and reperfusion, improved left ventricular end-diastolic pressure and relaxation time and prevented lung congestion in comparison to placebo. No anti-fibrotic effect was observed. Gene expression analysis revealed a change in remodeling genes comparable to the ACE inhibitor ramipril. In isolated cardiomyocytes lixisenatide reduced apoptosis and increased fractional shortening. Glucagon-like peptide-1 receptor (GLP1R) mRNA expression could not be detected in rat heart samples or isolated cardiomyocytes. Surprisingly, cardiomyocytes isolated from GLP-1 receptor knockout mice still responded to lixisenatide.

**Conclusions:**

In rodent models, lixisenatide reduced in an acute setting infarct-size and improved cardiac function when administered long-term after ischemia-reperfusion injury. GLP-1 receptor independent mechanisms contribute to the described cardioprotective effect of lixisenatide. Based in part on these preclinical findings patients with cardiac dysfunction are currently being recruited for a randomized, double-blind, placebo-controlled, multicenter study with lixisenatide.

**Trial registration:**

(ELIXA, ClinicalTrials.gov Identifier: NCT01147250)

## Background

Type 2 diabetes is a major health problem world-wide. In 2010, global prevalence of diabetes had reached 285 millions according to the International Diabetes Federation and is expected to increase by over 50% to 552 million by 2030 [1]. Disease progression is characterized by insulin resistance in association with relative insulin deficiency and hyperglycemia. Type 2 diabetes confers about a two-fold excess risk for a wide range of vascular diseases, independently from other conventional risk factors [2]. Available anti-diabetic drug classes have very limited or no documented benefit on cardiovascular outcome. Therefore, there is a high need for new anti-diabetic drugs that not only improve glycaemic control but also cardiovascular outcome in type 2 diabetes mellitus (T2DM) patients.

New therapies based on the incretin hormone glucagon-like peptide-1 (GLP-1) are currently tested in clinical studies on their ability not only to improve the metabolic dysfunction in T2DM patients but also to reduce cardiovascular risk. Additional GLP-1 effects beyond glycaemic control have been postulated based on the observation that the G-protein coupled receptor for GLP-1 (GLP1R) is expressed in many other organs and cell types including cardiovascular tissues (for review see [3,4]). GLP-1 infusion resulted in direct vascular relaxation assessed by flow-mediated vasodilation [5]. When added to standard therapy in patients with acute myocardial infarction and successful angioplasty, a 3d long infusion of GLP-1 was safe, well-tolerated and improved regional and global left ventricular function [6]. Chronic infusion of GLP-1 significantly improved left ventricular function, functional status, and quality of life in patients with severe heart failure [7]. Despite these promising cardiovascular effects, the therapeutic potential of GLP-1 is limited mainly because of its short half-life by rapid breakdown through dipeptidyl-peptidase-4 [8].

To overcome this hurdle exenatide and liraglutide have been introduced as more stable peptidic GLP-1 receptor agonists. Both share amino acid sequence homology to GLP-1 (53% and 97%) and display prolonged half-life in humans allowing twice daily or once-daily dosing. A third analog, lixisenatide is a new potent and selective peptidic GLP-1 receptor agonist for once daily s.c. injection currently in late stage clinical development. European market approval was recently granted under the trade name Lyxumia. In animal models of diabetes lixisenatide improved basal blood glucose and metabolic dysfunction with a rapid onset and sustained duration of action, prevented the deterioration of pancreatic responsiveness, delayed gastric emptying and reduced food intake [9]. Dose-dependent effects of lixisenatide in T2DM patients inadequately controlled with metformin were demonstrated in a randomized, double-blind, placebo-controlled trial [10].

Despite currently ongoing cardiovascular outcome studies in more than 26,000 T2DM patients directly treated with exenatide, liraglutide, or lixisenatide, basic mechanistic questions regarding the cardiac mode of action of these GLP-1 receptor agonists remain puzzling. All analogs have been tested in only a limited set of pre-clinical cardiovascular studies, and here with a strong focus on infarct size reduction and acute cardioprotection [11-13]. So far none of the GLP-1 analogs has been administered in chronic studies only after the onset of myocardial infarction. Hence the efficacy on cardiac remodeling beyond an acute anti-ischemic effect with infarct size reduction is not clear. Finally specificity of expression measurements of GLP1R in cardiac tissues has been recently challenged. Some GLP-1 peptide analogs exert GLP-1 receptor independent effects in the myocardium [14].

Here, we first investigated the acute effects of lixisenatide on acute infarct-size reduction in an isolated Langendorff heart preparation. In a second chronic rat study, with a transient ischemia setting designed to match closer to the clinical situation, lixisenatide administration was started clearly after the acute damage. A clinically established ACE inhibitor, ramipril, served as calibrator in this study protocol. In addition several mechanistic and cellular studies were performed to spread light on underlying signaling pathways and molecular mechanisms.

## Methods

All animal studies conformed to the German law for the protection of animal guidelines and the guide for the care and use of laboratory animals published by the US National Institutes of Health (NIH Publications No 85–23, revised 1996) as well as to Sanofi-Ethical Committee guidelines. Approval was granted by a local animal studies ethic review board.

### Specific materials

Ramipril and lixisenatide were synthesized in chemical departments of Sanofi.

The peptide sequence of lixisenatide is as follows: H-His-Gly-Glu-Gly-Thr-Phe-Thr-Ser-Asp-Leu-Ser-Lys-Gln-Met-Glu-Glu-Glu-Ala-Val-Arg-Leu-Phe-Ile-Glu-Trp-Leu-Lys-Asn-Gly-Gly-Pro-Ser-Ser-Gly-Ala-Pro-Pro-Ser-Lys-Lys-Lys-Lys-Lys-Lys-NH2.

For animal studies, lixisenatide was freshly dissolved in a sodium acetate buffer consisting of sodium acetate trihydrate (3.5 mg/mL), glycerol (85%, 18 mg/mL), L-methionine (3 mg/mL), and m-Cresol (2.7 mg/mL). Ramipril was mixed in chow at a concentration of 20.8 ppm resulting in a dose of 1 mg/kg/d. GLP-1 (7–36) amide and GLP-1 (9–36) amide were obtained from Bachem and used according to the manufacturer’s recommendations.

### Acute ischemia-reperfusion study in isolated rat hearts

Wistar rats (250-300 g) were heparinized (1000 U/kg) and then anaesthetized with pentobarbital-sodium (Narcoren®, 160 mg/kg i.m.). The heart was quickly excised and placed in ice-cold & filtered Krebs-Henseleit buffer consisting of 118 mM NaCl, 2.5 mM CaCl_2_, 4.7 mM KCL, 24.9 mM NaHCO_3_, 1.2 mM KH2PO_4_, 10 mM Glucose, 2 mM sodium pyruvate, and 1.6 mM MgSO_4_ adjusted to pH 7.5 and gassing with 95% O2/ 5% CO2. Following a stabilization period of 15 min, the left anterior descending artery (LAD) was occluded for 45 min, then re-opened and the hearts reperfused for 120 min. Lixisenatide (0.3 nM) was compared to placebo treatment in respective groups of n=10-11 hearts. Continuous treatments were started 10 min before occlusion till the end of the 120 min reperfusion period. During the entire experiment, cardiac hemodynamics were recorded, specifically left ventricular pressure (LVP), contractility (LVdP/dt_max_), relaxation (LVdP/dt_min_), coronary flow and heart rate. Finally, infarct size and area at risk of infarct determination was performed by Evans blue and triphenyltetrazolium chloride (TTC) staining. Quantification of stained slices was performed using the Morpho-Expert analysis software (Explora Nova, LaRochelle, France).

### Long-term study in rats after transient cardiac ischemia and reperfusion

Adult male Wistar rats (9 weeks old, 250-300 g, Harlan) were anesthetized with a mixture of Ketavet® (Ketamine, 66 mg/kg) and Domitor® (medetomidine hydrochloride, 0.090 mg/kg i.m.), intubated endotracheally and ventilated with a device (RUS-1301 Universeller Respirator, Föhr Medical Instruments GmbH, Germany). Body temperature was controlled and maintained at 37°C by an infrared bulb. The heart was accessed via left thoracotomy. The left coronary artery was isolated by using a 6–0 Prolene^TM^ suture (Ethicon®) with a tapered needle. The suture was tightened over a piece of PE-10 tubing to induce reversible ischemia for 30 min. Ischemia was accompanied by pale coloration of LV myocardium. Thereafter, the suture was released to start reperfusion. The thorax was closed with 2–0 Vicryl (Ethicon®) sutures, as well as the skin incision with 2–0 sutures. Anesthesia was neutralized by injection of Antisedan® (Atipamezolhydrochlorid, 0.5 mg/kg, i.m.). For pain relief Dipidolor® (Piritramid, 3 mg/kg, s.c.) was given.

Once the recovery was complete, the animal was returned to the rodent animal house facility. One day after surgery, animals were randomized into three treatment groups with at least 18 animals per group and consisting of I/R placebo, I/R ramipril (1 mg/kg/d pressed in chow) and I/R lixisenatide (10 μg/kg/d s.c.) over 10 weeks. A fourth group consisted of sham operated animals, without the myocardial ischemia-reperfusion procedure. After 10 weeks treatment, the animals were anesthetized with 100 mg/kg i.m. pentobarbital-sodium (Narcoren®). Left ventricular pressure (LVP), left ventricular end-diastolic pressure (LVedP), dP/dt_max_, dP/dt_min_, heart rate and tau Weiss were continuously recorded by a Millar tip catheter placed into the left ventricle. Finally the heart was arrested in diastole by infusion of a saturated potassium chloride solution. Hearts were rapidly excised and weighed (total heart weight). Thereafter atrial as well as non-cardiac tissues were carefully removed, left-ventricular (LV) and right-ventricular (RV) weight were measured and the left ventricle was kept for further evaluation of histology, fibrosis and gene expression measurements. Left ventricle containing the papillary muscles was fixed in buffered 4% paraformaldehyde and embedded in paraffin for histological evaluation. LV sections at the papillary muscles level were cut at 5 μm, deparaffinized, rehydrated and stained with picrosirius red to visualize interstitial and peri-vascular fibrosis. The percentage of the left ventricle stained for collagen was calculated as the ratio of picrosirius-red positively stained area over total tissue area using Morpho Expert image analysis software (Explora Nova, La Rochelle, France).

### Biomarker assessment in the long-term study

A set of biomarkers were assessed using plasma and serum obtained at the end of the long-term ischemia reperfusion study. Serum levels of cholesterol, triglycerides, creatinine, urea, glucose and ACE-activity were determined on a Hitachi 912 analyzer, using the respective Roche clinical chemistry kits for human diagnostics.

Serum BNP 32 as a biomarker for rat heart failure was determined by a commercial ELISA (Phoenix Pharmaceuticals, Inc. Karlsruhe, Germany). Serum insulin concentrations were determined using a commercial rat ELISA kit (Mercodia, Upsala, Sweden).

### Gene expression studies using left ventricular sections of the long-term study

RNA was isolated from formalin-fixed and paraffin-embedded tissues. Using a sharp razor blade each paraffin-embedded LV block was divided into half separating the free LV wall part from the septal wall part and 16 consecutively divided slices (10 μm) were collected into Eppendorf tubes. A specific RNA isolation kit was used according to the manufacturer’s instructions (RecoverAll Total Nucleic Acid Isolation Kit, #AM1975, Life technologies, Darmstadt, Germany). Reverse transcription into cDNA (10μL RNA, approximately 10 ng/μl) was performed using a high-capacity kit (cat# 4374966, Life technologies). Then a quantitative pre-amplification step consisting of 14 cycles was included using 4μL cDNA solutions, 4μL target specific primer pool, and 8μL TaqMan PreAmp MasterMix (cat# 4391128, Life technologies). The results pre-amplified solutions were diluted with water and mixed with TaqMan Universal PCR Master Mix (cat# 4369016, cat# 4374966, Life technologies) according to the manufacturer’s instruction. In case of single PCR reaction independent wells were filled with pre-amplified DNA, specific primers and master mix. In addition, ports of 384 well format micro-fluidic cards were filled with 100 μl sample solution, briefly centrifuged twice for 1,200 rpm and sealed. Each micro-fluidic card contained 8× 96 genes (93 target genes and 3 housekeeping genes) listed in the Additional file 1. Real-time PCR (40 cycles) were performed using a ViiA7 cycler (Life technologies).

### Isolation of rodent cardiomyocytes

Ventricular cardiomyocytes were isolated from 250-300 g male Sprague-Dawley rats as previously described [15] with some modifications. During retrograde perfusion of excised hearts Liberase-IV as digesting enzyme was used (8.75 Wünsch units, Roche, Mannheim, Germany). The enzyme was re-circulated for 30 min in Joklik Medium (Biochrom, Berlin, Germany) additionally buffered with HEPES (15 mM). Thereafter atria were removed, and ventricles minced and cut into pieces in Powell’s Medium (145 mM NaCl, 2.6 mM KCl, 1.2 mM KH_2_PO_4_, 1.2 mM MgSO_4_, 15 mM HEPES, 11 mM glucose, pH 7.4) containing 20 μM CaCl_2_ and 15 mM 2,3-butanedioneoxime-diacetylmonoxime (BDM, Fluka Chemie, Germany). After filtering through a nylon mesh, cells were centrifuged at 30 × g for 4 min. Extracellular calcium was stepwise increased (200 μM, 400 μM, 1 mM) by overall 3 centrifugation steps (30 × g for 4 min). Finally, the isolated cardiomyocytes were suspended in fetal calf serum free Joklik medium with 1 mM CaCl_2_ and 5 mM BDM and plated at a density of 1.5×10^4^ rod-shaped cells per cm^2^ cultivation area. Two hours after plating, cultures were washed with basic culture medium consisting of HEPES-buffered Joklik medium with 5 mM creatinine, 2 mM L-carnithine and 5 mM taurine, 100 IU/ml penicillin, 100 μg/ml streptomycin, 100 μM ascorbic acid and cytosine-ß-D-arabinofuranoside (10 μM) as further supplements. A similar isolation protocol was used for isolation of mouse cardiomyocytes from either GLP1R knockout mice or age-matched CD1 mice serving as wildtype control. Four mouse hearts were perfused in parallel and isolates combined thereafter.

### Measurement of apoptosis and fractional shortening analysis in cardiomyocytes

In order to determine apoptosis, caspase-3/7 and cell viability were determined in rat cardiomyocytes incubated in 35 mm culture dishes in HEPES-buffered Joklik medium with either insulin or lixisenatide for 18 h. Caspase-3/7 activity and cell viability were determined in independent dishes using chemiluminiscence kits (G8092/3 and G7571, Promega, Madison, USA). Contractile responses of single cardiomyocytes were measured by an electro-optical monitoring system as described in detail elsewhere [16] with some minor modifications. Briefly, cells were measured after a resting period of 24 h in 35 mm culture dishes (Falcon, type 3001) in modified Tyrode’s solution (145 mM NaCl, 2.6 mM KCl, 1.2 mM KH_2_PO_4_, 1.2 mM MgSO_4_, 1.0 mM CaCl_2_ 10 mM HEPES, 10 mM Glucose, pH 7.4). Single cells were subjected to a biphasic rectangular voltage ramp from −50 to 50 V with 0.5 ms duration at a frequency of 0.5 Hz. When the contraction amplitude reached stability, four contraction cycles were recorded and determined via standard software. In each batch of isolated cardiomyocytes at least 18 cells were measured for each condition and the results finally averaged. Each experiment was repeated twice on independent cell isolations.

### Overall data analysis and statistics

Data from each experiment and study were carefully analyzed using SAS (version 8.2) for SUN 4 via interface software EverStat V6.0. First, data were analyzed for normality and for homogeneous variances (Levene). In case of Gaussian distributions, ANOVA was employed. In case of heterogeneous variances and/or non-Gaussian distribution, a Kruskal-Wallis test was used followed by the Kruskal-Wallis multiple comparisons test versus Placebo. P-values <0.05 were regarded as statistically significant. Data are presented as mean ± SEM.

Standard gene expressions analysis was performed based on the Δc(t) method. Standardization was related to a geometric mean value of all housekeeping genes based on the bestkeeper algorithm [17]. A gene expression level was set as undefined if no amplification was achieved at maximum cycle time (c(t) > 40). Array Studio (Omicsoft, V6.1) was used for micro-fluidic card PCR analysis and subsequent principle component analysis, hierarchical clustering and generation of heatmaps. Missing data was imputed using k-nearest neighbors, method with the missing value replaced by the mean of the 5 nearest neighbors. For the 2D hierarchical clustering, Pearson dissimilarity was selected for the distance metric and complete linkage was used for the tree joining method. The heatmap used standardized values for visualization.

## Results

### Acute ischemia reperfusion injury in isolated rat hearts

**Figure 1:**
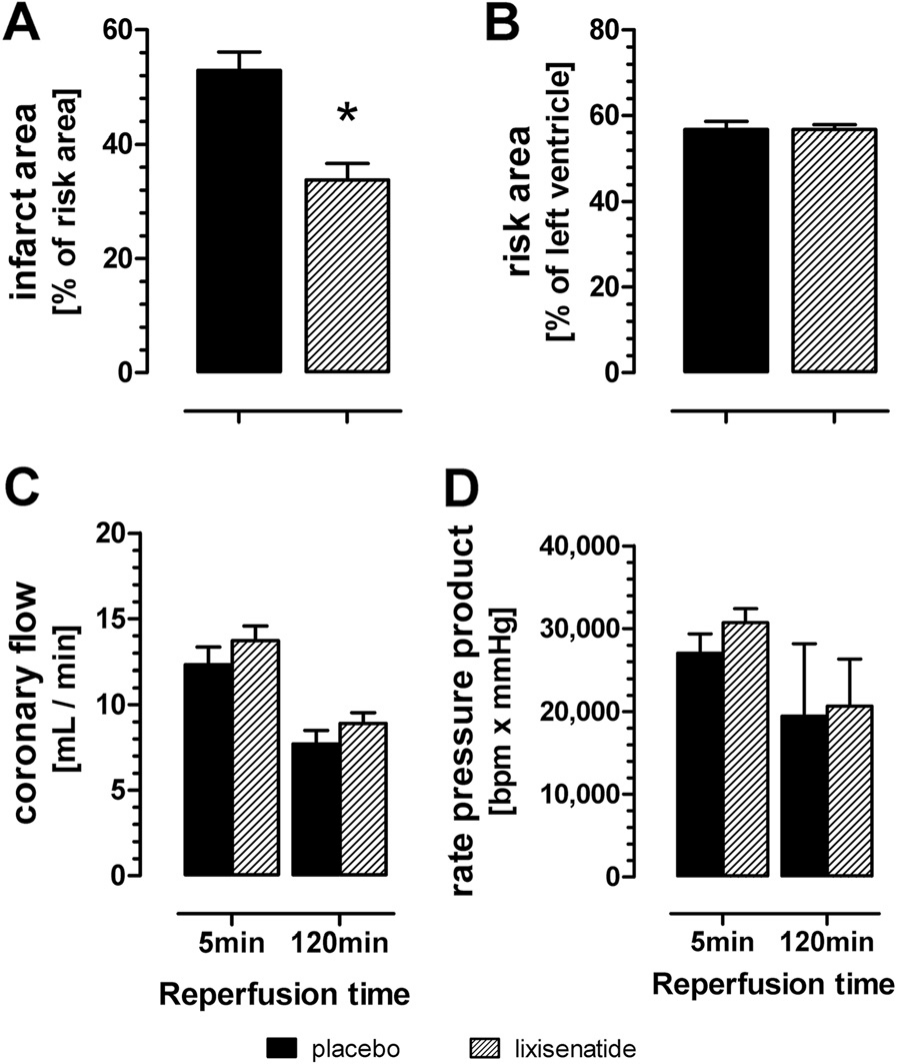
**Effect of lixisenatide on infarct size and cardiac function in isolated rat hearts subjected to ischemia (45 min) and reperfusion (120 min).** (**A**) infarct to risk area ratio, (**B**) risk area, and (**C**, **D**) selected coronary flow and rate pressure product immediately after onset of reperfusion (5 min) and at the end of reperfusion (120 min). ^*)^ denotes p < 0.05 versus placebo.

The LAD coronary artery was occluded for 45 minutes followed by reperfusion for 120 minutes. Treatment with lixisenatide significantly reduced infarct-size when starting 10 min prior to end of ischemia (Figure 1). Infarct area in placebo hearts was 162 ± 12 mm^2^, and in lixisenatide treated hearts 98 ± 9 (mean values ± SEM, n=10-11, p < 0.05). There were no differences between the different treatment groups regarding left ventricular total area (543 ± 16 mm^2^ for placebo versus 505 ± 7 mm^2^ for lixisenatide treatment) left ventricular risk area (306 ± 9 mm^2^ for placebo versus 288 ± 5 mm^2^ for lixisenatide treatment). Overall, the ratio of infarct area to area at risk was reduced by lixisenatide by 36% when compared to placebo treatment (Figure 1A). The rate pressure product (LVP x HR) and coronary flow were not altered during the reperfusion period as shown for 5 and 120 minutes (Figure 1B and C).

### Long-term injury induced by a transient ischemia reperfusion

In order to reveal beneficial effects of lixisenatide long-term treatment on cardiac remodeling, a study in rats with transient ischemia and 10 weeks reperfusion was performed. The major ischemia-reperfusion damage occurs within the first day after myocardial infarction. By randomization of infarcted animals to different treatments after the acute damage period, we excluded potential effects on infarct size as confounding factor. Main intention of the long-term study was to prove efficacy on post myocardial infarction induced remodeling and not acute anti-ischemic efficacy.

**Figure 2:**
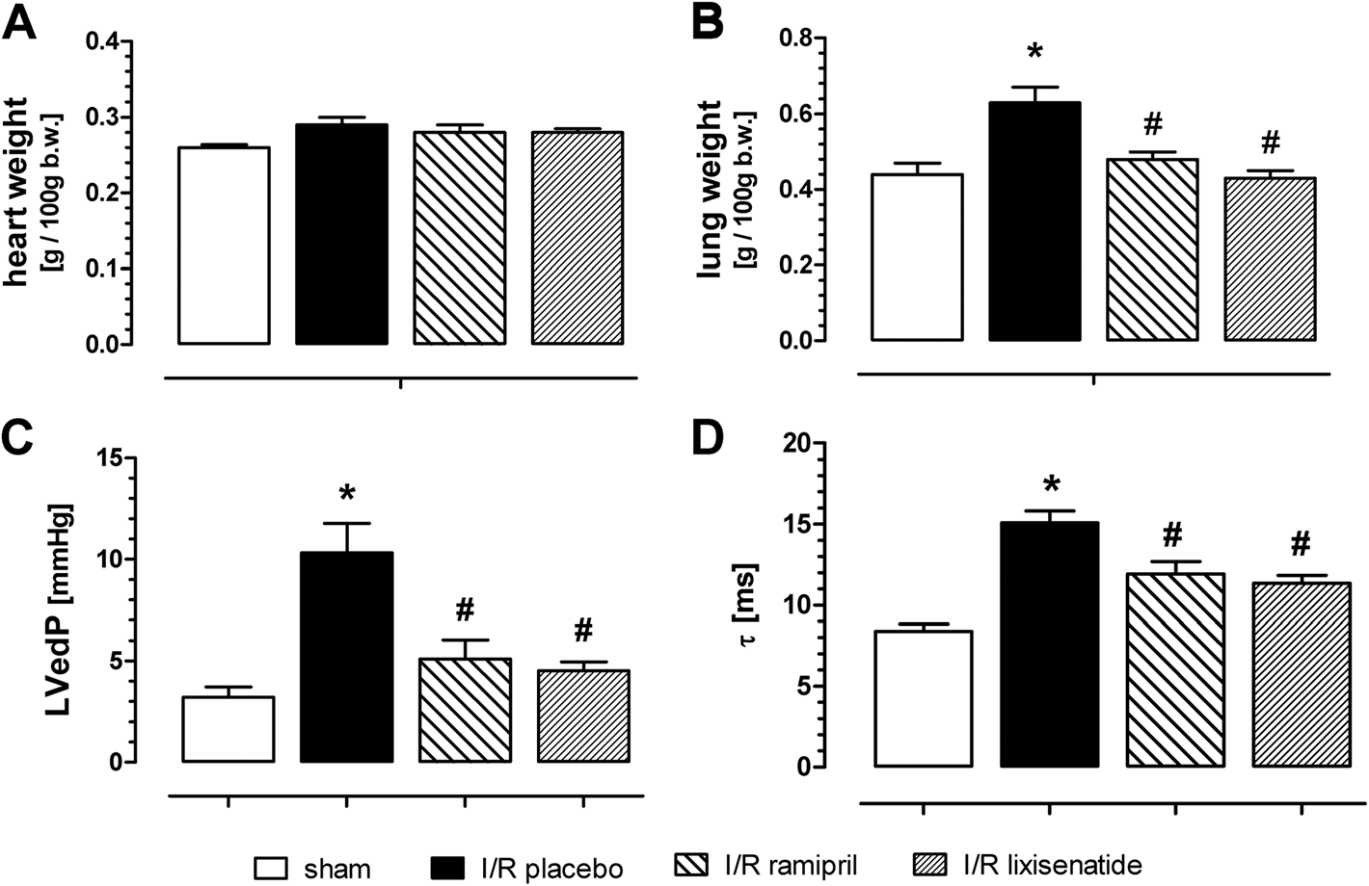
**Effect of long-term treatment with lixisenatide or ramipril after transient ischemia-reperfusion injury:** Heart weight. (**A**), lung weight (**B**), left-ventricular end diastolic pressure (LVedP, **C**) and relaxation time (tau Weiss, **D**). ^*)^ denotes p < 0.05 versus sham and ^#)^ p < 0.05 versus placebo.

Body weight but not food consumption was slightly lower in rats treated for 10 weeks with lixisenatide (10 μg/kg/d) or the ACE Inhibitor ramipril (1 mg/kg/d p.o.) compared to I/R placebo group (see Table 1). Ischemia and long-term reperfusion resulted in cardio-dynamic impairment that was attenuated by treatment with either lixisenatide or ramipril. Indeed, increased left ventricular end-diastolic pressure as a measure for impaired diastolic function, tau Weiss as a measure for myocardial relaxation, and lung weight as a measure of congestion, were all significantly improved towards sham values for the treatment groups (Figure 2).

**Table 1:** Effect of long-term treatment with ramipril (I/R ramipril) and lixisenatide (I/R lixisenatide) after transient ischemia reperfusion injury

	**Sham**	**I/R Placebo**	**I/R ramipril (1 mg/kg/d p.o.)**	**I/R lixisenatide (10 μg/kg/d s.c.)**
Body Weight (g)	441 ± 9	432 ± 9	403 ± 7*	390 ± 6*
Food consumption (mg/kg/d)	73 ± 3	78 ± 2	72 ± 5	79 ± 4
Water consumption (mL/kg/d)	116 ± 3	120 ± 2	168 ± 5*	120 ± 2
Plasma BNP (pg/mL)	112 ± 5*	178 ± 14	113 ± 6*	110 ± 7*
Plasma glucose (mmol/L)	8.8 ± 0.2	8.8 ±0.3	8.6 ± 0.3	8.0 ± 0.4
Plasma triglyceride (mmol/L)	0.61 ± 0.09	0.49 ± 0.05	0.44 ± 0.04	0.47 ± 0.03
Plasma ACE activity [U/L]	186 ± 17	206 ± 37	51 ± 11*	192 ± 10
LVP (mmHg)	118 ± 5*	106 ± 3	97 ± 2*	105 ± 3
LVedP (mmHg)	3.22 ± 0.49*	10.32 ± 1.45	5.11 ± 0.93*	4.52 ± 0.43*
dP/dt max (mmHg/sec)	6695 ± 268*	5023 ± 210	4724 ± 197	5136 ± 243
dP/dt min (mmHg/sec)	−6877 ± 373*	−4463 ± 206	−4499 ± 184	−4683 ± 278
Heart Rate (beats/min)	324 ± 8	316 ± 6	327 ± 7	308 ± 8
tau Weiss (msec)	8.39 ± 0.45*	15.08 ± 0.75	11.93 ± 0.76*	11.37 ± 0.47*
Heart Weight (g/100 g BW)	0.26 ± 0.01*	0.29 ± 0.01	0.28 ± 0.01	0.28 ± 0.01
Cardiac expansion index	0.29 ± 0.02	0.28 ± 0.04	0.23 ± 0.05	0.30 ± 0.05
Lung Weight (g/100 g BW)	0.44 ± 0.03*	0.63 ± 0.05	0.44 ± 0.02*	0.43 ± 0.02*
Liver Weight (g/100 g BW)	3.31 ± 0.04	3.23 ± 0.06	3.12 ± 0.05	3.02 ± 0.16
Right Kidney Weight (g/100 g BW)	0.30 ± 0.01	0.30 ± 0.01	0.35 ± 0.01*	0.30 ± 0.01

Abbreviations used are brain natriuretic peptide (BNP), left ventricular pressure (LVP), left ventricular end-diastolic pressure (LVedP), myocardial contractility/relaxation (dP/dt_max_, dP/dt_min_), heart rate, relaxation time (tau Weiss). Mean values ± SEM are shown. *) indicates p < 0.05 versus the placebo group (I/R injury without treatment).

**Figure 3:**
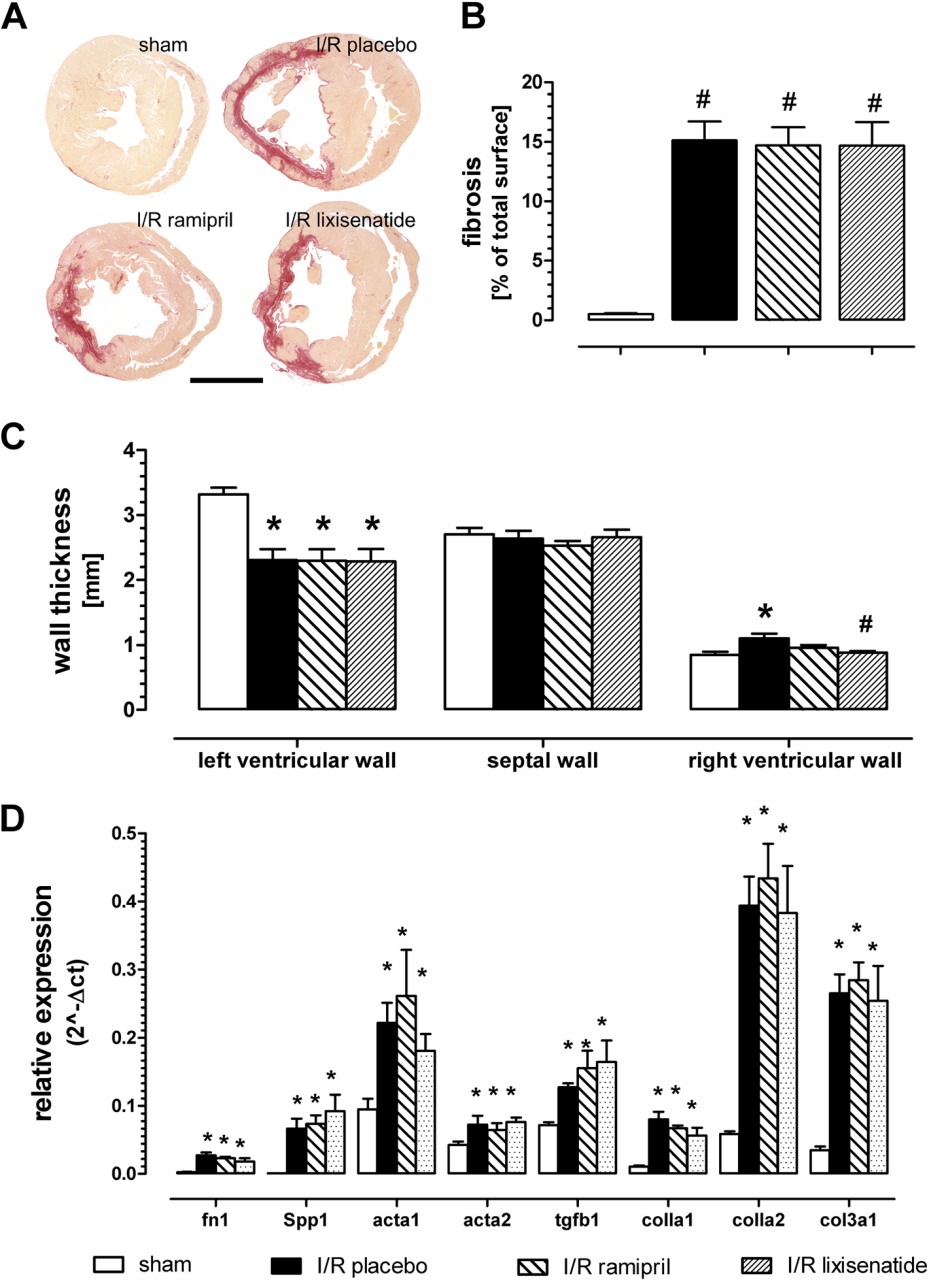
**Effect of long-term treatment with either lixisenatide or ramipril after transient ischemia-reperfusion injury on heart morphology.** (**A**) Picrosirius red staining at the papillary muscle level of representative animals, Bar = 5 mm; (**B**) quantification of left ventricular fibrotic to total area; (**C**) thickness of the left ventricular, septal and right ventricular wall; and (**D**) gene expression analysis by realtime PCR of selected fibrosis genes. ^*)^ denotes p < 0.05 versus sham and ^#)^ p < 0.05 versus placebo.

Increased serum levels of brain natriuretic peptide (BNP) were normalized in lixisenatide or ramipril treated animals. No effect on plasma glucose or triglyceride was observed. ACE activity was blocked in the ramipril but not the lixisenatide group (see Table 1). A slight but non-significant increase in heart weight could be observed in I/R placebo animals (Figure 2A). No overall cardiac dilatation could be seen using the histologically defined expansion index introduced by Hochmann and Choo (Table 1) [18]. Left-ventricular wall thickness was reduced by ischemia-reperfusion and not significantly modified by treatment groups (Figure 3C). Septal wall thickness was not altered between any groups. Slightly increased right ventricular wall thickness was normalized to sham value by lixisenatide treatment.

A significant increase in left ventricular fibrosis was observed by the ischemia-perfusion procedure assessed by picrosirius red staining (Figure 3A and B). Neither lixisenatide nor ramipril treatment resulted in a decrease in fibrosis. At the end of the chronic study, we analyzed the expression of a number of selected genes in slices taken from infarcted and non-infarcted areas of the hearts. In the infarct area, remodeling genes like osteopontin and collagen isoforms were up-regulated by the ischemia reperfusion damage. In accordance with the histological staining expression of those genes was not modulated by either ramipril or lixisenatide (Figure 3D).

**Figure 4:**
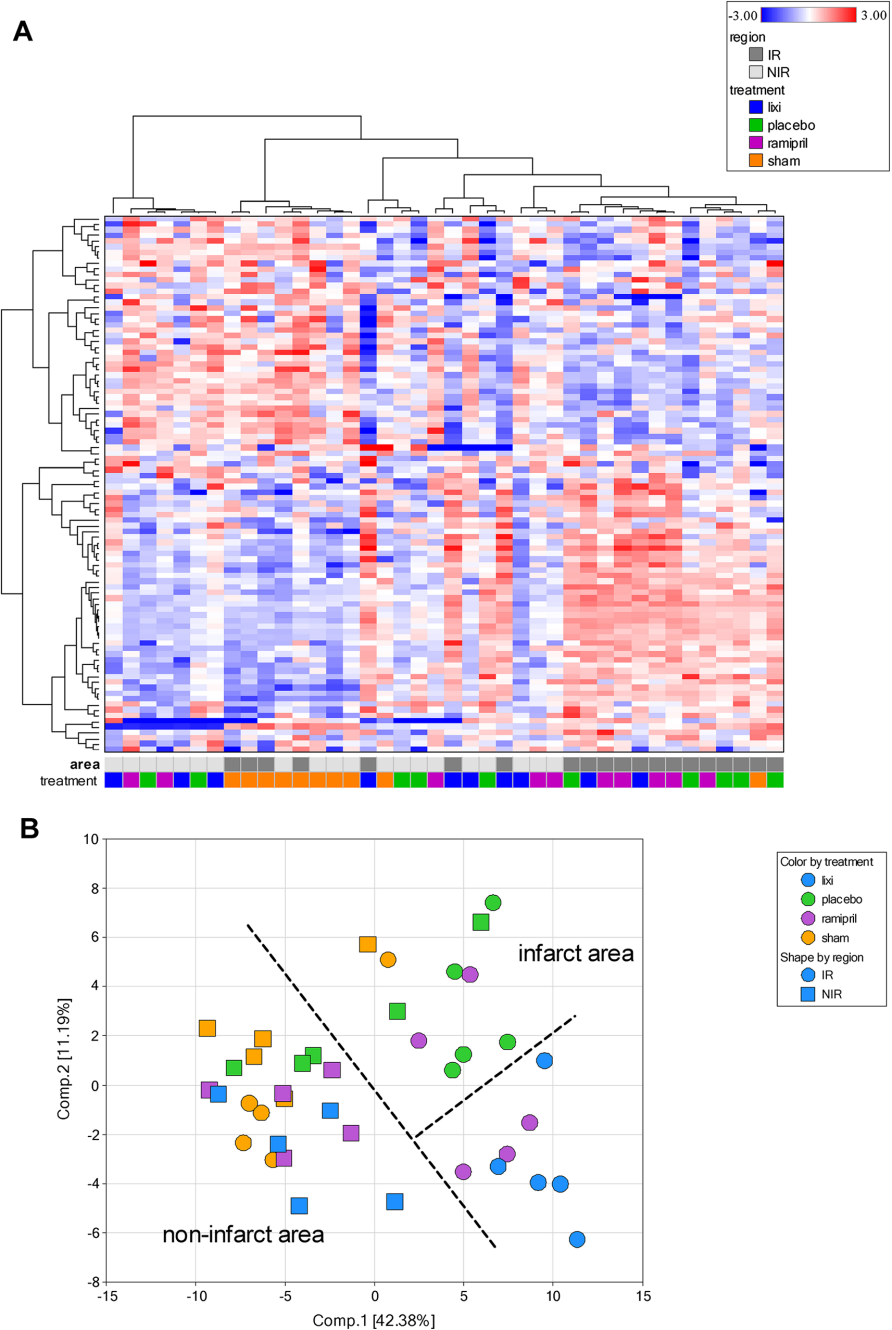
**Gene expression patterns 10 weeks after ischemia reperfusion injury performed on all samples and comparing non-infarct area versus infarct area.** (**A**) Determination of 92 representative genes followed by 2 dimensional hierarchical clustering and visualization as heatmap. Each sample forms a column and each gene forms a row with the heatmap intensities standardized within each gene. Overall 5 independent samples were measured for each group and two different tissue areas (infarct and non-infarct ventricular tissue) resulting in a final number of 40 samples. (**B**) Principle component analysis (PCA) of the same data reflecting the total expression changes into two uncorrelated principal components.

In order to reveal a more global reaction pattern, expression of 93 specific genes and 3 housekeeping genes was quantified in RNA isolated from the tissue slices (n=5 per treatment group and area) by micro-fluidic card PCR. The 93 genes were pre-selected based on published cardiac remodeling data and covered apoptosis, autophagy, remodeling, and inflammation pathways. A heat map visualization of these data indicated differential patterning (Figure 4A). First, the majority of sham treated samples were linked together in one major cluster (left part of the heatmap). Another major cluster included infarct area samples and here two subclusters from either placebo or lixisenatide/ramipril treatment (right part of the heatmap). A principle component analysis (PCA) was performed on the gene expression data to reduce the complexity of 93 genes expressions within one sample into independent principal major components (Figure 4B). Two major clusters became visible. Most of the non-infarct regions samples clustered together with nearly all sham samples. Gene expression in the infarct regions formed another cluster with 2 subcluster separating all lixisenatide and 3 of 5 ramipril samples from placebo, respectively. We further performed a 2 way ANOVA analysis on differentially regulated individual genes. Additional file 2 lists all genes with a p-value of p<0.05 comparing the different treatments and areas. Lixisenatide and ramipril treatment resulted in the infarct area in a similar up- or down regulation of only three genes by a factor greater than 1.5 fold. To reveal lixisenatide specific gene regulations unbiased Affymetrix microarray measurements maybe a next useful step.

### Effects in isolated rodent cardiomyocytes

**Figure 5:**
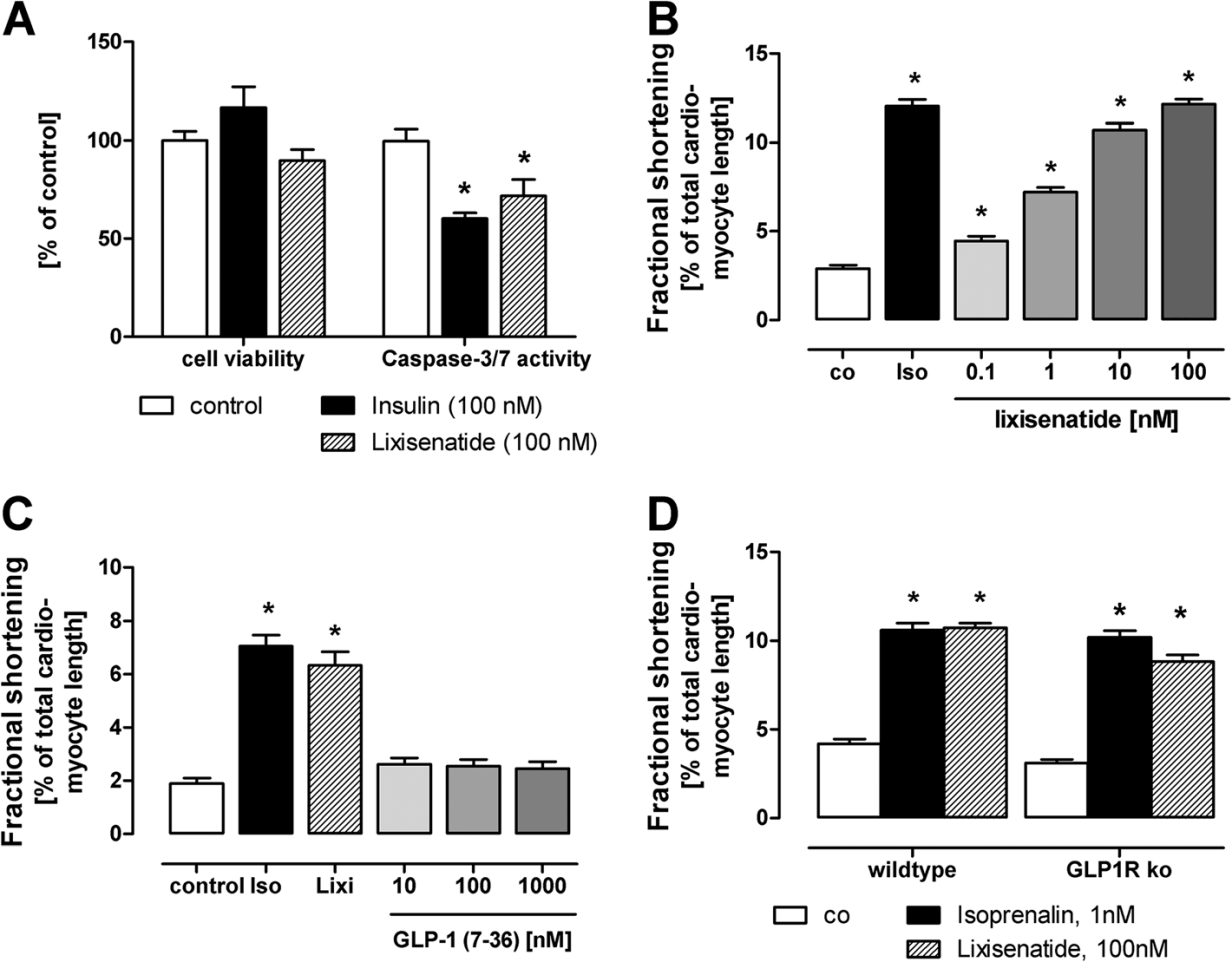
**Effect of lixisenatide treatment on apoptosis and contractility of isolated cardiomyocytes.** (**A**) Caspase-3/7 activity is reduced in cardiomyocytes incubated with either insulin or lixisenatide (each at 100 nM). (**B**, **C**) Lixisenatide (Lixi) but not GLP-1 (6–36)-amide increases fractional shortening. Isoprenaline (Iso, 1nM) served as positive control. (**D**). Cardiomyocytes isolated from GLP-1 receptor knockout mice respond to lixisenatide. Mean values and SEM are given. In the fractional shortening experiments, n > 18 single cardiomyocytes were measured for each group.

To better understand the beneficial effects of lixisenatide, we performed experiments with lixisenatide on isolated cardiomyocytes. Longer incubation of rat cardiomyocytes with either lixisenatide or insulin resulted in a decrease in caspase-3/7 activity indicating a reduction in cardiomyocyte apoptosis (Figure 5A). A concentration-dependent increase in fractional shortening was observed by stimulation with lixisenatide that was comparable to adrenergic stimulation with isoprenaline (Figure 5B). This functional response was partly reversed by brief pre-incubation with the PI-3-kinase inhibitor wortmannin (see Additional file 3: Figure S3). Other GLP-1 receptor agonist like exenatide-4 also resulted in increased fractional shortening (see Additional file 3: Figure S3). However, the endogenous GLP-1 receptor agonist GLP-1 (7–36)-amide did not elicit significant increase even at highest concentrations (Figure 5C). Cardiomyocytes isolated from both GLP-1 receptor knockout mice and respective wild-type animals (CD1) responded to lixisenatide (Figure 5D) with an increase in fractional shortening.

### Expression of genes involved in the GLP-1-incretin axis

**Figure 6:**
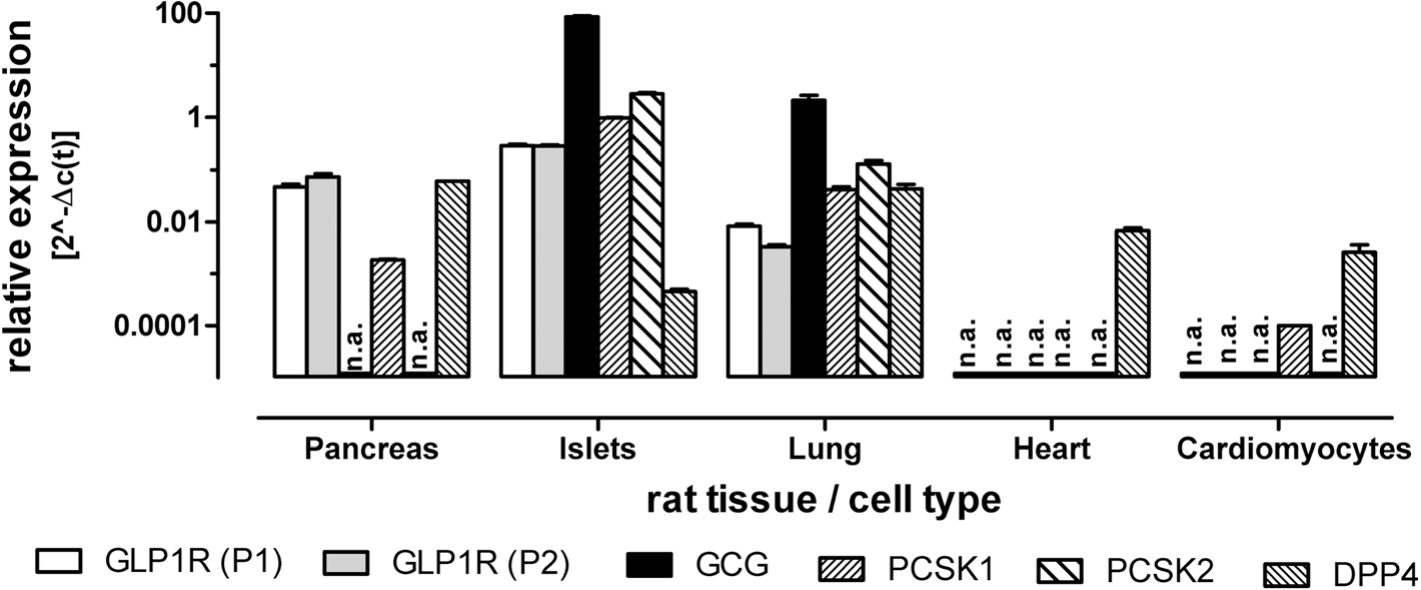
**Expression of 5 genes involved in GLP-1 incretin biology in different rat tissues assessed by quantitative Taqman PCR.** Two biologically different samples were analyzed and averaged (shown are mean values and SD). The geometric mean of three housekeeping genes (GAPDH, Actb and B2M) was used to normalize expression of specific genes using the formula 2^-Δc(t). For detection of GLP1R two different specific Taqman primer assays were used spanning different part of the receptor mRNA. GLP1R (P1) denotes Rn00562406_m1 and detect exon 3–4. GLP1R (P2) is the label for Rn01640381_m1 which detects a sequence spanning exon 8–9. The term n.a. indicates that no specific amplification could be achieved at the maximum cycle number (n=40).

A local GLP-1 release may exist in the heart, potentially competing with lixisenatide on the GLP-1 receptor. Therefore we analyzed tissues for the expression of the GLP-1 precursor peptide, glucagon (GCG), and converting and degrading proteases, PCSK1, PCSK2 and DPP4 (Figure 6). Neither GCG, nor PCSK1 or PCSK2 expression could be observed in cardiac samples ruling out the existence of a local GLP-1 generation.

In parallel we determined the expression of GLP-1 receptor using two different specific Taqman primer assays spanning different exon/intron within the known rat sequence (Figure 6). Use of both primers assays indicated a similar strong expression in pancreas, isolated pancreatic islets and lung, but failed to amplify specifically any signal from cardiac tissue and cardiomyocytes. It seems that all cardiac tissues lack components of a local incretin system with DPP-IV being the exception.

## Discussion

Since the early 1990s GLP-1 peptides and analogs have been investigated for treating T2DM because of their ability to enhance glucose-dependent insulin secretion. In addition, GLP-1 agonists are hypothesized to have effects on the cardiovascular system beyond glycaemic control, which may be exploited for therapeutic benefit [19,20].

Cardiovascular disease is the leading cause of death in patients with type 2 diabetes. In a recent prospective cohort study of one million U.S. adults, diabetes was associated with a twofold increase in the risk of death from ischemic heart disease [21]. Concerns about the cardiovascular safety of anti-diabetic drugs resulted in the FDA recommendation that the CV risk should be more thoroughly addressed during drug development [22]. As a consequence of this new guideline cardiovascular outcome studies are currently ongoing in more than 26,000 type 2 diabetic patients directly treated with GLP-1 receptor agonists and more than 40,000 treated with a DPP-IV inhibitors which also may act indirectly via GLP-1. The overall goal of our study was to demonstrate in a pre-clinical set of models cardioprotective effects of the GLP-1 analog lixisenatide, building a further fundament for clinical testing in patients with reduced cardiac function.

In an acute model of global cardiac ischemia-reperfusion injury, lixisenatide treatment during the last 10 minutes of ischemia and during the whole reperfusion period significantly reduced infarct size. The observed cardioprotective effect was not associated with a significant change in cardiac hemodynamics, as assessed by rate-pressure product RPP (LVDP x HR), and particularly coronary flow. The results described here with lixisenatide are consistent to similar results in previous studies with another GLP1R agonist, exenatide [11]. Ischemia reperfusion injury is a multifaceted damage, comprising effects on cardiomyocytes function and death and endothelial function. Due to the lack of effect on coronary flow by lixisenatide, we assume a direct effect on cardiomyocyte function and survival. Sonne and co-workers found that GLP-1 (9–36) amide, which is the first breakdown fragment of GLP-1 (7–36) amide but not a GLP-1 receptor agonist, did not reduce infarct size but however increased functional recovery of ischemia hearts [11]. Based on these finding the authors proposed that beside the known GLP-1 receptor another hitherto unidentified receptor could be responsible for effects of GLP-1 like peptides in rat hearts.

Only a limited numbers of pre-clinical studies have investigated so far effects of GLP-1 peptides on long-term consequences after myocardial ischemia and reperfusion. Using liraglutide, Noyan-Ashraf and coworkers demonstrated cardioprotection after myocardial infarction in diabetic and non-diabetic mice [13]. Liu and coworkers treated rats two weeks after myocardial infarction with either GLP-1 (7–36) or the exenatide analog AC3174 and noticed improved cardiac function and morphology [23]. In contrast to those previous studies, we performed only a transient and not permanent ligation of the left coronary artery in our rat model in order to obtain results closer to the clinical situation in which almost all patients with myocardial infarction are reperfused. The transient ischemia reperfusion protocol resulted in only moderate changes of systolic function after a long-term recovery period. LVP and dp/dt_max_ were slightly reduced in the ischemia reperfusion group versus sham treated animals. Ramipril but not lixisenatide normalized LVP but not dp/dt_max_. Major differences were observed for the active treatments regarding diastolic function, in particular on left ventricular end diastolic pressure (LVedP) and the relaxation time tau Weiss. These functional improvements occur despite lack of effect on increased cardiac fibrosis assessed by specific morphological staining and gene expression analysis. In addition, heart weight did not significantly differ between the various groups ruling out strong effects on hypertrophy of cardiomyocytes as potential mode of action. Moreover, we noticed no effects on plasma glucose and insulin by lixisenatide treatment in this non-diabetic model. Hence, indirect effects on heart metabolism via glucose uptake may not sufficiently explain the observed improvement in cardiac function.

A broader gene expression pattern of genes involved in rat cardiac remodeling delivered two major findings. First, the myocardial infarction is a major driver of gene expression changes and drug effects in the non-infarct regions of infarcted hearts are rather moderate. Second, lixisenatide and the ACE inhibitor ramipril are similar in their reaction pattern, indicating activation of more common protective signaling pathways for both treatments. A principal component analysis supported these conclusions. An ANOVA analysis provided a few number of genes differentially regulated by lixisenatide versus placebo in the infarct area (see Additional file 2).

Interestingly, lixisenatide was equipotent to GLP-1 and other GLP-1 receptor analogues on reducing infarct size in perfused isolated rat hearts. In contrast, lixisenatide at higher concentrations, but not GLP-1, improved fractional shortening in isolated cardiomyocytes. This might suggest that lixisenatide displays acute cardioprotection via the GLP-1 receptor, while effects of lixisenatide on contractility are mediated by a different signalling pathway. In line with our findings, Vila Petroff et al. showed the lack of contractility-promoting effect of GLP-1 in isolated cardiomyocytes [24]. We supplemented their findings by demonstrating that even very high concentrations of the GLP-1 peptide, not amenable to rapid degradation by dipeptidyl-peptidase-IV (DPP4), were ineffective. Finally, when performing a similar set of experiments on cardiomyocytes from GLP-1 receptor knockout mice, a robust response to lixisenatide remained suggesting a GLP-1 receptor independent effect lixisenatide on the contractility response.

Lixisenatide displayed beneficial effects in our rodent models, but GLP-1 receptor mRNA was not detectable without ambiguity. Two different PCR Taqman probes, located to different parts of the rat gene, did not reveal abundant expression of the GLP-1 receptor in rat hearts and cardiomyocytes, but indicated a strong expression in other organs like rat pancreas. Overall, controversial data on the expression of the GLP-1 receptor in mammalian cardiac tissues exists. GLP-1 receptor mRNA expression in human heart samples was detected by an RNA protection assay [25]. In contrast, using autoradiography with a radio-labeled GLP-1, no staining was detected in human heart despite several other organs being positive for this ligand [26]. An antibody against the GLP-1 receptor stained in mouse heart several cell types [27]. Later it was shown that available antibodies against the GLP-1 receptor display strong cross-reactivity in cells not expressing the GLP-1 receptor [28]. Hence, immunostainings should be carefully considered as long as specificity is not clearly demonstrated. Simultaneously to immunostainings, Ban *et al.* detected mRNA for the GLP1-receptor, using an endpoint PCR with a high number of amplification cycles followed by specific hybridization [27]. This methodology does not rule out a very low expression level in the samples. Bullock and co-workers used the RNAse protection assay, a more quantitative but less sensitive methodology, to assess mRNA distribution in rat tissues for the GLP-1 receptor. They could not confirm expression in the rat heart in contrast to other positive tissues like pancreatic tissues [29].

Currently, we cannot exclude that a GLP1R variant is expressed in the heart which is not detectable using the two different PCR primer assays. In addition, a normal GLP1R may be expressed in rodent hearts in other cell types than cardiomyocytes with overall low abundance, e.g. in resident or invading immune cells. Alternatively, another receptor, not related in its primary structure to the GLPR1 may exist in the rat heart that is responsive to lixisenatide and other GLP-1 analogs, mediating the cardioprotection seen in our studies. Clearly, further work needs to be invested here, e.g. testing of lixisenatide and related GLP-1 like analogs on ligand efficacy of a broad panel of receptors.

The pre-clinical effects described here provide a rationale for further clinical testing of lixisenatide in patients at cardiovascular risk. In a first randomized, double-blind, placebo-controlled, multicenter study patients are currently being recruited (ELIXA, ClinicalTrials.gov Identifier: NCT01147250). The primary objective of this study with approximately 6,000 patients is to demonstrate that lixisenatide can reduce cardiovascular morbidity and mortality compared to placebo in type 2 diabetic patients who recently experienced an acute coronary syndrome event.

## Conclusions

We could demonstrate that lixisenatide induced cardioprotection in short- and long-term rat models of ischemia-reperfusion ischemia. Most probably direct effects on cardiomyocytes independent of the GLP-1 receptor improving function and reducing apoptosis explain best the cardiac efficacy of this peptidic GLP-1 receptor analog. The mechanism of the lixisenatide mediated cardioprotection warrants further investigations.

## Competing interests

Dominik Linz has no conflict of interests. Jochen Huber has accepted a research position at Boehringer Ingelheim, Biberach, Germany and has no conflicts of interest. All other authors are employees of Sanofi’s R&D Diabetes Division and involved in pre-clinical research and identification of new approaches in Diabetes. Lixisenatide is currently being developed by Sanofi for the treatment of T2DM patients.

## Authors’ contributions

PW carried out cardiomyocyte experiments and gene expression measurements and provided a major contribution the design of the manuscript its coordination and its writing. TH performed all histochemistry and image analysis and provided a major contribution in the animal data coordination. WL and DL carried out the long-term ischemia reperfusion rat study. JH carried out the isolated heart experiments. DC performed bioinformatics data handling and statistical analysis. SH, UW and HR participated in the design and coordination of all animal studies and helped to draft the manuscript. All authors read and approved the final manuscript.

## Supplementary Material

Additional file 1 List of specific Taqman primers used in the micro-fluidic card analysis. Click here for file

Additional file 2 **Expression analysis of 93 selected genes at the end of chronic treatments after MI.** Normalized abundance data are given for each treatment and two different heart regions. Slices of the septum served to determine expression in no-infarct-region. Slices from the infarcted left ventricular region contain mainly infarcted area. This file also contains a comparision of up- and downregulated genes between treatments and compared to the placebo group. Only genes are shown with a p-value of p<0.05. The upper part consisted of infarcted area, the lower of non-infarcted area. Upregulation <1.5 fold is indicated by an underlying orange colour. Down-regulations >1.5 fold are shaded blue. Click here for file

Additional file 3 **(A) Effects of co-inbuation of wortmannin on lixisenatide induced cardiomyocyte contractility.** (B) Comparision of different GLP-1 like peptides (each at 100 nM) on cardiomyocyte contractility. ^*)^ denotes p<0.05 versus control; ^#)^ denotes p<0.05 versus lixisenatide treatment. Click here for file
