# Supplementary figures and images for: Cardioprotective effects of lixisenatide in rat myocardial ischemia-reperfusion injury studies
Source: J Transl Med. 2013 Mar 28;11:84. doi: 10.1186/1479-5876-11-84 (PMC3637243; doi:10.1186/1479-5876-11-84)

**A**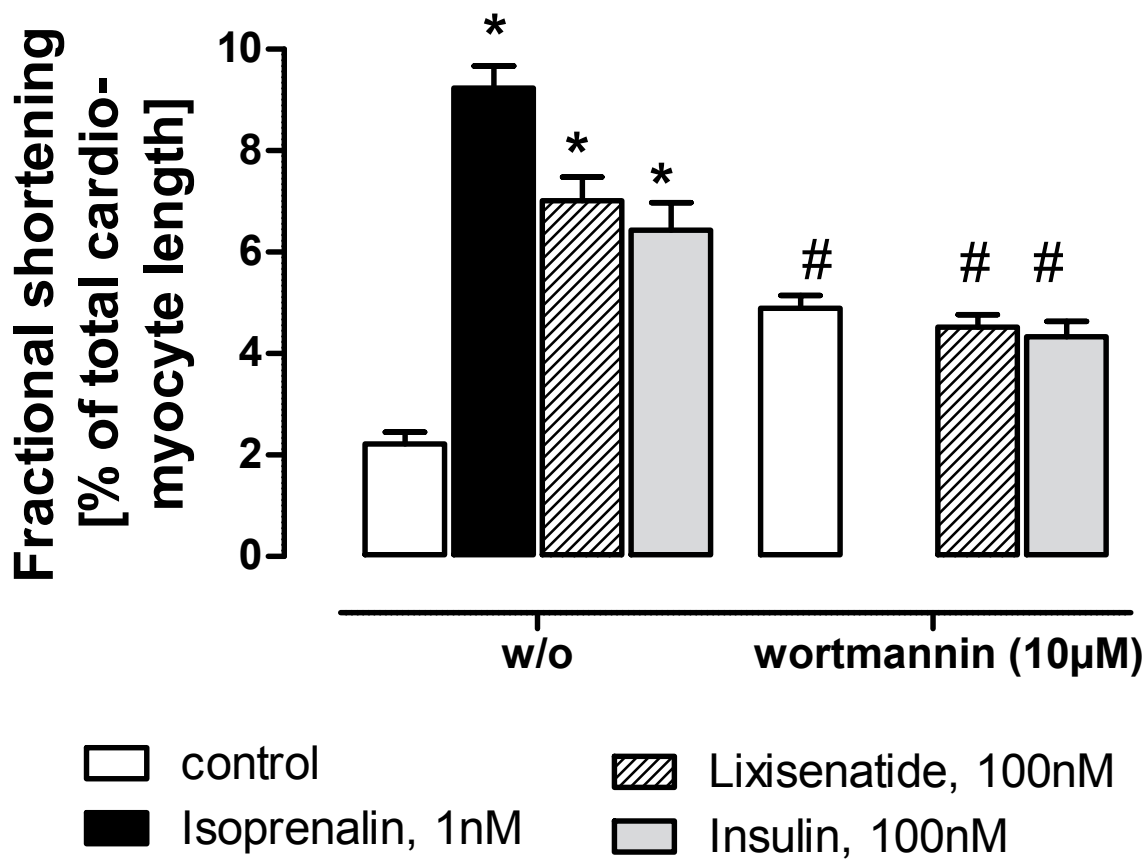**B**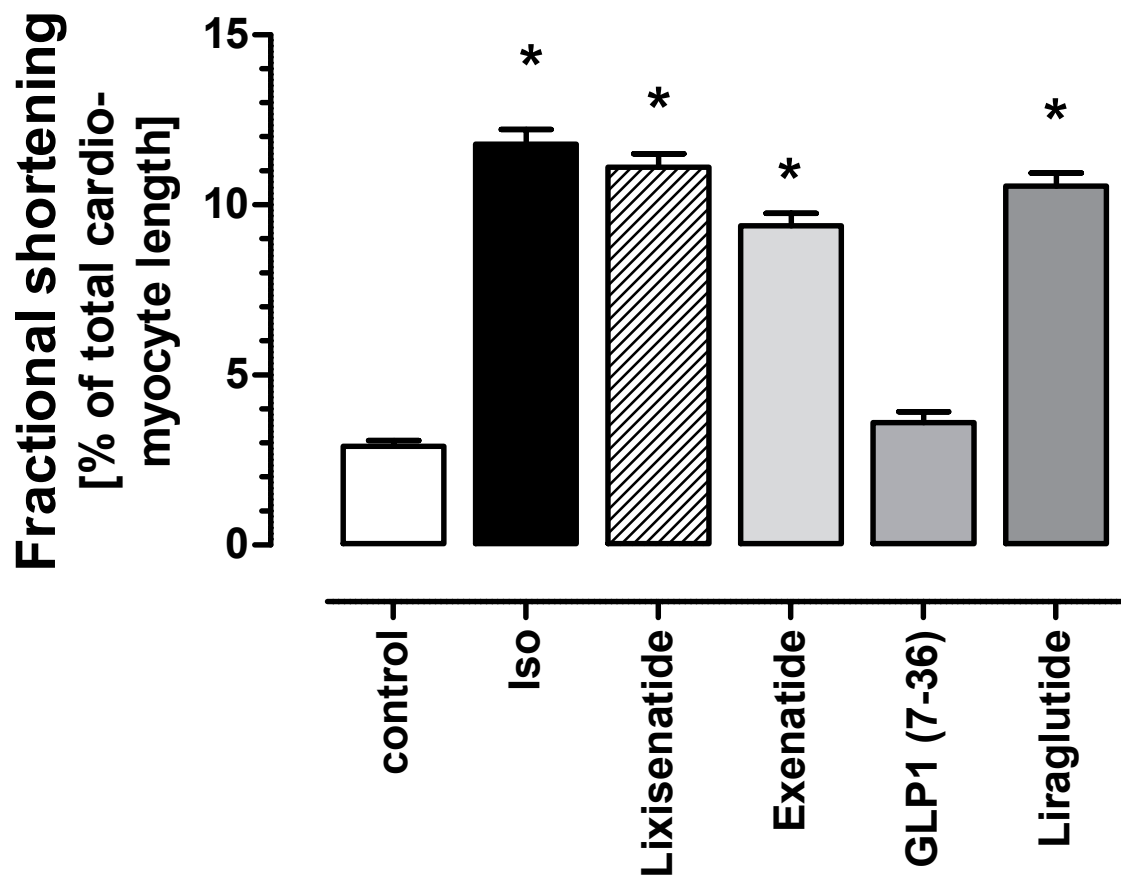

Supplement: Additional file 3 — (A) Effects of co-inbuation of wortmannin on lixisenatide induced cardiomyocyte contractility. (B) Comparision of different GLP-1 like peptides (each at 100 nM) on cardiomyocyte contractility. *) denotes p<0.05 versus control; #) denotes p<0.05 versus lixisenatide treatment. [file 1479-5876-11-84-S3.pdf]
